# Our Summer at Maplewood, Chap. IX

**Published:** 1875-10

**Authors:** 


### CHAPTER IX.

### IN THE ORCHARD.

We were in the orchard looking for apples to eat, to bake, and to make into pies; and with baskets well filled with Sweets, and Codlings, and Queens, Emmeline and I sat under the shade of a tree, while Alice wandered farther on in search of fruit that was rosier-cheeked, or more golden, than any we had gathered.

"Did it ever occur to you, Kate," said Emmeline, breaking the silence into which we had fallen, "that there is a universal disposition among all classes of people to wait on the future, and make little account of the present? Most of us fail to enjoy the present, we are so occupied in preparing for the future. In consequence of this feeling we store our closets with preserved fruits, which are in a measure dead fruits, the life, the pure air and sunshine, having been crushed and cooked out of them, in the process of canning and preserving. When winter comes our cellars are filled with fragrant, delicious apples; so full of goodness that they make the atmosphere, even of a close damp cellar, redolent of sweetness. But, in our blind stupidity, we pass by, with a careless word of commendation, the very prince of fruits, the apple, leaving it to wither and decay, while we feed on the husks of the past, the cherries, and berries, and peaches, and plums, that we have canned, and preserved, and pickled. How foolish it does seem to neglect the apple, when it is in its very prime, through the fall and winter months, for these fussed-up favorites, which are far less nutritious; and which we ate far too sparingly in their natural state, and while in season, during the sultry summer-time."

"Yes, Emmeline, it often seems to me that in caring for the future we waste much of the present. Do you know, by the way, that some wise people say that apples contain all forms or kinds of nutriment essential to sustain and nourish our bodies, and that we can live and thrive upon apples alone, if we use them cooked and uncooked, as food? For the past week I've been thinking about the best methods of canning, preserving, and cooking fruits, and the very thoughts you express have occurred to me. If people were wise enough to eat plentifully of the different kinds of fruits in their proper season, there would be comparatively little need of canning and preserving, in many sections of our country."

"You may be correct in your views, Kate; but since people will "can" and preserve fruits for some generations yet, I hope you will write upon the subject and tell them how to do it according to the best methods. Many persons care little for cooked apples, I think, for the simple reason that they are generally cooked so wretchedly. Sometimes we get them half-baked, and at others we have them in a wishy-washy, watery sauce, so overdosed with sugar and nutmeg, or other heavy spices, that they nauseate delicate stomachs."

"Thank you, Emmeline. You give me a hint for my book: *Never spice fruits lest you destroy their flavor.* I always detested nutmeg, allspice, and cinnamon, in apple-pies. I now see the reason. Rich spices and delicate flavored fruits form an unsuitable, and inartistic combination. Heavy spices belong legitimately to heavy sweetness, such as cake, custard, and pumpkin-pie. But the fine delicate flavor of fruits, should be preserved as perfectly as possible; and for this reason they should never be cooked in tin, or stirred with a metal spoon, less pure than silver. A wooden spoon or spatula is best for the purpose. These are the directions I shall give: if it is desirable to add to the flavor of the fruit you are cooking, add the flavor of another fruit. For instance, flavor apples with either pine-apple, strawberry, raspberry, quince, lemon, or orange, &c. Even the perfume of flowers, like rose-water, may add to the deliciousness of a dish of fruit. But to bury roses in cake, seems as inappropriate and unnatural as to deaden fruit with spices. In cooking fruits of all sorts, whether for present or future use, aim to preserve the flavor of the fruit as far as possible; and to this end, avoid all contact with tin or base metal, and all needless exposure to the air. Cook as soon as possible after the fruit is in proper condition. Cook in small quantities. Simmer gently, instead of boiling rapidly. The flavor of some fruits is preserved better by canning them without sugar. Peaches and blackberries are much finer flavored when canned without sugar. At the time of opening and serving, sugar can be added, if desired. Currants and raspberries, when made into jams and jellies, should be simmered until nearly cooked, before the sugar is added. Now, the conveniences for canning fruits are so perfect, it is not necessary to add sugar for the purpose of preserving, but only to render palatable. All fruits will keep perfectly without sugar, when properly canned. They should be put up boiling hot, in air-tight glass cans; and, unless placed in a dark closet, should be wrapped in thick brown (or dark) paper, to exclude the light, which changes the color of some fruits, and injures the flavor of others. Strawberries, and perhaps some other very acid fruits, are best when sugar is added at the time of cooking. Emmeline, have you ever seen in any cook-book a recipe for making mushes or jellies of fruit by the addition of arrow-root, corn-starch, or wheat flour?"

"No, I never heard of such a thing until you served us those delicious strawberry, raspberry, and blackberry mushes, as you called them. And you have never yet given us the method of preparing them. Will you repeat it for my benefit?"

"To a quart of berries, fresh or canned, add a pint of water, and sugar to taste. Boil slightly, just enough to cook the fruit; and, when nearly done, add corn-starch, arrow-root, or wheat flour, wet with cold water, to thicken the juice, and form a mush or jelly of the fruit. Serve cold, with sweet cream."

"Kate, I never ate a more delicate, delicious dessert, than these fruit mushes, and they alone ought to render your book popular. What a charming dish for children and invalids, either strawberry, raspberry, or blackberry mush must make! I am surprised I never heard of fruit-mush before this summer."

"Emmeline, do you know I think currant jam very much nicer than currant jelly? It has a fresher, better flavor, which I presume is on account of its not being beaten and bruised, and then squeezed through a rag. For jam, bruise the fruit in an earthen bowl, just enough to break the skins and set the juice free. Put in a preserving-kettle. Simmer gently for twenty minutes, skimming carefully, after it begins to boil. At the expiration of the twenty minutes, add three-fourths of a pound of white sugar to each pint of crushed currants. Simmer two or three minutes longer, and put in glass cans or jars. One part red raspberries to four parts currants, changes, and, for many persons, improves the flavor."

"Kate, as your skill is to be tested to-day in making my favorite among pies, I shall, when we go into the house, read you what Beecher, in 'Eyes and Ears,' says on the subject of apple-pie."

When our pie was placed on the table at dinner, Emmeline got her book, and read:—

"'There is, for example, one made without under-crust, in a deep plate, and the apples laid in, in full quarters; or the apples being stewed are beaten to a mush, and seasoned, and put between the double paste; or they are sliced thin and cooked entirely within the covers; or they are put, without seasoning, into their bed, and when baked, the upper lid is raised, and the butter, nutmeg, cinnamon, and sugar, are added; the whole well mixed, and the crust returned as if nothing had happened. But oh, be careful of the paste! Let it not be like putty; nor rush to the other extreme, and make it so flaky that one holds his breath while eating for fear of blowing it away. Let it not be plain as bread, nor yet rich like cake. Aim at that glorious medium in which it is tender, without being too fugaciously flaky; short, without being too short; a mild, sapid, brittle thing, that lies upon the tongue, so as to let the apple strike through and touch the papillæ with a mere affluent flavor. But this, like all high art, must be a thing of inspiration or instinct. A true cook will understand us, and we care not if others do not!

"'Do not suppose that we limit the apple-pie to the kinds and methods enumerated. Its capacity in variation is endless, and every diversity discovers some new charm or flavor. It will accept almost every flavor of every spice. And yet nothing is so fatal to the rare and higher graces of apple-pie as inconsiderate, vulgar spicing. It is not meant to be a mere vehicle for the exhibition of these spices, in their own natures; it is a glorious unity in which sugar gives up its nature as sugar, and butter ceases to be butter, and each flavorsome spice gladly vanishes from its own full nature, that all of them, by a common death, may rise into the new life of apple-pie! Not that apple is longer apple! *It*, too, is transformed; and the final pie, though born of apple, sugar, butter, nutmeg, cinnamon, lemon, is like some of these, but the compound ideal of them all, refined, purified, and by fire fixed in blissful perfection.'"

"Now," remarked Emmeline, "let us see if this is equal to H. W. B.'s ideal pie!"

As she pressed the upper crust gently with the pie-knife the merest drop of candied juice appeared between its parted lips; and, although she lifted a piece to another plate with a quick, dexterous movement, a spoon was needed to dip from the pie-dish some juice, unavoidably spilled in the operation. Helping each of us in a similar manner, Emmeline ate a minute in silence, and then said, with a satisfied air,—

"Had brother Beecher eaten one of your pies, Kate, before he wrote that essay on apple-pie, he would not for the world have mentioned nutmeg, allspice, or cinnamon, in connection with apple-pie. He would have known they were of the earth, earthy, mere "inconsiderate, vulgar spicing"; good enough, perhaps, for uncultivated or depraved tastes; but not like this, hinting of orange-groves and fairer climes,—a combination of flavors and fragrance unknown to common mortals, who have never enjoyed such a perfect pie as the one now before us. Do tell us how this was fashioned, created, built up into such completeness?"

"Then, I suppose you wish to know, in the first place, how the crust is made?"

"Certainly. That is the body or flesh of a pie. The fruit is the heart, soul, and spirit. I wish to know how the crust and the fruit are compounded; how dough and apple are metamorphosed into a perfect apple-pie."

"To make pie-crust, one pound of flour, a quarter pound of lard, a quarter pound of butter, a glass of cold water, a cool pantry or room, and quick movements, are essential. Place half the flour in a little heap on the molding-board. Cut the lard into, and through it, until quite fine. Add cold water, and stir with the knife. When wet and quite soft, scrape from the board to one side. Sift from the other half of the flour upon the board, till well covered. Lay the dough on it. Roll it out, but be careful not to get it too thin. Spread half the butter over it, and then cover the butter with flour. Fold, and roll out. Put on the remaining butter, and cover with flour. Fold, and roll quite thin. Sift flour lightly on it. Fold, or roll up, and lay it aside. Take a little more than a fourth of the whole for one crust. Flour the board and dough, and roll to the size required. Place upon the tin or dish, shaping to the same, and cut off around the edge with a sharp knife. Fill the pie with quarters; or, if the apples are large, with eighths, of tart apples. Roll an upper crust, and lay it over the apples. Trim it around the edges as before, but do not pinch the two crusts together. If you wish to ornament the crust let the cuts or marks be upon the upper surface only. Do not cut through so as to make holes at which the steam can escape. Place the pie in a hot oven, so that the paste will bake quickly. When thoroughly baked, if the apples are not perfectly cooked, which can be easily ascertained by lifting the upper crust at one side and peeping in, lessen the heat, or transfer to a cooler oven, until done. If necessary, you can protect the crust with paper, from becoming too brown."

"Don't you put water in to cook the apples?" interrupted Alice.

"No. The apples are sufficiently juicy to cook themselves, if the steam and juice are kept confined in the pie, as they will be, if no holes are cut or pricked in the upper crust. While the pie is baking put in a cup or small stew-pan, the sugar required for it, two or three ounces if the apples are tart, as they should be, a small piece of butter, and the juice of an orange, with a very little of the outside grated, if you like it. Melt all together; and, when the pie comes from the oven, slip it from the tin or dish on which it was baked, to the warmed plate on which it is to be served. Lift off the upper crust. Pour your seasoning over the apple, distributing it evenly; replace the upper crust, press it gently, and, in one hour the pie is at its best. A pie baked in this manner does not, however, lose its goodness within an hour. The next day, and the day after even, you will find it something more than "a corpse of an apple-pie." For four pies, if the apples are not very tart, and you like a rich pie, use the juice of one lemon with that of two oranges and a little grated peel, adding sugar in proportion. Most delicious apple-pies are made by using one grated pine-apple to four pies. Place the grated pine-apple with the butter and sugar in the stew-pan, and heat to boiling, then add as before, laying it lightly and evenly over the apple, which is permeated by the mixture, without being stirred."

"Mamma," said Alice, "you have neard of cambric tea. Did you ever hear of linen pie?"

"Oh yes. I have heard people call hot water, with cream and sugar to taste, by that name. They say Horace Greeley used to consider cambric tea a delightful beverage. I drink it sometimes myself; but I believe I have never tasted a linen pie. How is it made?"

"Cousin Kate makes it in this way. Instead of filling her pie-crust with apple, or other fruit, she fills it with pieces of old white linen, and bakes. Meanwhile, on the range she prepares the fruit, if it is desirable to cook it; and, when the crust is baked, she slips it on a plate, lifts off the upper crust, takes out the rags, and fills their place with the juicy fruit, which couldn't possibly be baked in the pie, without losing a large portion of the juice, and spoiling the looks, as well as the taste of the pie. At dinner every one who doesn't understand the trick wonders how so juicy a pie was so perfectly baked. And no one can imagine how the upper crust was baked so rich a brown, without being stained with juice, and the under crust so crisp, without being soaked or heavy. To the uninitiated it is no doubt as great a marvel as the apple inside the dumpling was to the bewildered king, who asked, amazed, 'How got the apple in?'"

"There is another advantage, Alice," I said, "in baking these linen pies. Several crusts may be baked and put away for a day or two unfilled; and an hour or two before serving, if placed for a few minutes in a hot oven, or until warmed through, and then filled with fresh fruit they can scarcely be distinguished from freshly baked pies. These linen pies are just the things to fill with strawberries or other fruit which is good uncooked."

"To change the subject a little, Emmeline, I agree fully with the remark you made this morning, that few persons care for cooked apples, because they are, as a general thing, so wretchedly cooked. And, I think, for the same reason vegetables are almost discarded, in many families. Nearly all classes of people crave green food; and it seems to me the craving ought to be gratified, by their making fruits, berries, and vegetables, a part of their regular daily diet. When people eat freely of well-cooked vegetables, fruits, and berries, at their regular meals, as they do other articles of diet, they have little disposition to indulge in the injurious habit of eating between meals; while, on the other hand, in families where such things are seldom served at meal-time, men, women, and children, hanker after unripe fruit, even; and render themselves liable to all manner of diseases by eating green apples, half-ripe cherries and berries, raw turnips, &c.

"In one of her letters to me, Mrs. Rose writes: 'I never put sugar *on* berries for tea. It spoils the shape of the berries to let them lie in or under sugar.' Now, there is no doubt it spoils the shape of berries to sugar them; but, for most persons, it improves the flavor to sprinkle them with sugar ten or fifteen minutes before the berries are served. No one disputes the orthodoxy of Mr. Beecher's opinions on gustatory matters, however much they may differ from him on theological subjects, and *he* approves of mashing them, even. He says: 'Put ripe berries in a dish, add a little *cold water*, break them down with a spoon to a jelly, adding just enough sugar and water to make them half liquid, and you shall find many another dish less delicious.' This disposition to preserve the shape of articles of food is quite prevalent among housewives; and the taste of many a dish is sacrificed, in order that it may make a handsome appearance on the table. Fruits, grains, and vegetables, are too often purposely sent to table half cooked, so that the shape may not be spoiled. In all such cases the eye is gratified at the expense of the stomach. The idea that these articles, when ready for the table, should present the same form and appearance as when uncooked, is, in my judgment, a very erroneous one. Food of every kind should be thoroughly cooked—even though in the operation all form and comeliness be destroyed—as it is then more palatable and nutritious to healthy stomachs. There is, however, a vast difference between thorough cooking and too much cooking. Food is as effectually spoiled by too much as by too little cooking; and any one wishing to excel in culinary science must note carefully the distinction, and strike the happy medium. If vegetables were properly cooked, and fruits and berries were properly prepared for the table, they could scarcely fail to be always acceptable to the average appetite, and would soon force themselves into general use. I may be too enthusiastic; but I look forward hopefully to the time when they will be so cooked and prepared; and when cartloads of vegetables will be consumed where an occasional mess is now deemed sufficient; and when fruits, cooked and uncooked, will be considered as essential to a meal as bread or meat.

"The apple, although it contains so many elements of goodness, is one of our most abused fruits. As a general rule, it is shabbily treated in every process of cooking it is compelled to undergo. It is badly baked, and wretchedly stewed. It is diluted with water, or dosed with sugar, or doctored with spices, until all the original flavor is lost or spoiled. But, in spite of all this bad usage, the apple has retained a place in the culinary department, and is destined, as soon as we learn how to cook it properly, to grow into universal favor, and become a prominent article of diet at every well-ordered table."

"Many persons," observed Emmeline, "are of the opinion that only sweet apples are good for baking. I, however, like a tart apple, when baked, much better than I do a sweet one. Which do you prefer, Cousin Kate? And what is your method of baking apples?"

"I very much prefer sour apples. And this, I think, is the best way of baking them: Take large, juicy, sour apples. Pare them, and remove the cores, leaving the apples whole; place them in a deep earthen dish; add to them one tablespoonful of water; put them in a hot oven, and bake until perfectly soft and tender. A few minutes before removing from the oven, sprinkle them lightly with white sugar. They will then brown richly, and have a delicious flavor. Serve warm, hot, or cold, according to taste. Most people soon grow so fond of them as to eat them with their meats and vegetables; and also to take them, garnished with sugar and cream, for dessert, in preference to either pudding or pie.

"To bake apples whole, remove all imperfect spots or specks; wash and wipe dry; pierce the skins in sundry places with a fork; place in an earthen pan or dish, and bake in a hot oven. An apple baked quickly, with all the heat it can bear, is very different from one baked in a moderate oven. The former is spicy and full of spirit, while the latter tastes as if all life had been worried out of it by slow torture.

"Apple-sauce, as usually made, is scarcely fit to eat; yet, when properly prepared, it is one of the most delicious dishes of which I have any knowledge. Apples should always be stewed in a porcelain-lined kettle,—never in a vessel made of tin. Very little water should be added to them; and they should not be stirred while cooking, if possible to avoid it. They should be covered closely, and cooked quickly; and should be watched all the time while cooking, lest they should burn. When done, the sugar required should be put into an earthen bowl, and the apple poured over it, and covered closely until served. Stewed apple that is continually stirred while cooking, is not spicy and high-flavored, like that quickly and quietly cooked. As a usual thing, too much sugar is used; so much, in fact, that all apple flavor is lost in the heavy sweetness. To preserve the perfect flavor of stewed apples, or apple-sauce, great care is required in the judicious use of sugar or spices.

"The Health Food Company, 137 Eighth Street, New York, have a delicate preparation which they call 'Pomarius.' It is jelly-like, yet it contains no gelatine; it is pleasantly sweet, while containing no added sugar, and is tart without acidity. It is delicious as an adjunct to meats, or eaten with bread or any of the mushes, and proves very grateful to the stomach and very useful as a digester of heavier foods. It is simply the juice of sound, ripe, tart, juicy apples; not the kind known in the market as 'cooking apples,'

but the choicest samples to be found. These nice apples are crushed, the juice is expressed and filtered, and is then evaporated by new and delicate processes. Some of these processes are as critical as the most important known to the chemist's art. The product is, as may be supposed, beautiful, cleanly, and wholesome, while being pervaded with the pleasantest of fruit odors. I believe it is a substance which will speedily be adopted into every refined family, as soon as known. It keeps, uncovered, without fermentation or deterioration."

"It must be the perfection of fruit jellies," said Emmeline, "and would, I should think, render palatable all farinacious preparations, which are apt to be a little insipid to tastes impaired by spices and sauces. By the way, I have somewhere eaten a pudding made of apples and tapioca, that I thought very nice. Have you ever made it?"

"Oh yes," I replied. "Such a pudding when properly made, and served cold, is a very delicate dessert. I think to serve it warm, however, as it is often served, is as poor taste as to serve a custard-pudding warm. To make tapioca-pudding, use five measures of cold water to one measure of tapioca. Let it soak over night, or for several hours; then cook it until it looks clear and transparent. While the tapioca is cooking, pare and core some large greenings, or other tart apples. Leave the apples whole. Place them close together in a deep earthen dish, and pour the tapioca over them. Bake thoroughly, until the apples brown over the top. When quite cold serve with sugar and sweet cream. I have no doubt that this new article, 'Pomarius,' would prove very appetizing, if treated in a somewhat similar way."

After dinner Emmeline and I were on the veranda, chatting in an indolent, indifferent way, on various topics, when Alice appeared with the small gilt-edged volume, and interrupted us by saying,—

"Mamma, I've brought this 'Memoir of Helen Douglas,' to read you some extracts. I have always detested memoirs, and thought them the stupidest of books ever invented, calculated only to bore amiable and well-disposed people. But this differs in that respect from the usual memoir. I have marked some passages that I think suggestive as well as beautiful. Shall I read them to you?"

"By all means, if Cousin Kate doesn't object," was Emmeline's reply.

"Cousin Kate," I said, "will be delighted to hear them."

Alice then read:—

"'To the average mind the idea of immortal life presents itself simply as a continuation, after death, of the life we have lived in this world, extending through Eternity: free, however, from all the cares and sorrows, all the pains and infirmities, of this mortal existence. But if we retain our personal consciousness in Eternity, will we not retain also the facilities that were essential to our development in Time? Or will all our emotions, passions, feelings, be sloughed off with our flesh, by Death? How will we recognize beyond the grave those whom we loved so fondly here? Will my lost Helen meet me in years hence the same gentle being that went from my sorrowing gaze?

Or will she be changed, re-created, formed anew? How shall I know her

> '—— in the land that keeps
> The disembodied spirits of the dead'?

Or what assurance have I that our love shall not change with a change cf existence? Oh, God, why am I tormented with these painful questionings?'

——

"'Whence do we come, or whither do we go? No one can answer satisfactorily. It is an unsolvable riddle. All guess-work, speculation. Joy and happiness are mere illusions — the flickering sunshine of a moment. Pain is a reality. The only tangible thing in life is suffering, sorrow.'

——

"'Since Helen died all things seem changed. When she was alive I looked eagerly forward. The future seemed bright and beckoning. So full of great hopes, of grand possibilities. My hours were crowded with work. The days were too short to satisfy me, I had so much to do. Now I dare not look backward, I care not to look forward. To do either seems like peering into the gloom of a winter's night. The present is full of heaviness. I can not set myself to any task, and thus escape from my great grief, for nothing now seems worth the doing.'

——

"'Will the days ever come when I can realize—

> ''Tis better to have loved and lost,
> Than never to have loved at all'?

Life is poor and barren without love; but how painfully desolate it becomes after the loss of the one loved better than all else on earth! I was the victim of unrest before love entered, and took possession of my heart; nevertheless, I was negatively happy. Now, my days and nights are filled to overflowing with sorrow. A weary life is mine to bear.'

——

"'Now that Helen has been torn from me, after so short a season of unalloyed happiness, I tremble at the thought of annihilation, and refuse to believe the gloomy doctrine. Yet, I can find no satisfying evidence of the soul's immortality,—nothing to convince my mind, beyond a doubt, that there is a world and a life beyond the tomb. Love, however, uproots disbelief. Selfishness is more satisfying than reason. The yearning to be again with Helen dispels doubt, and overturns anguish. We must, we will, meet again. But is this life to be projected into the next, with all its hates as well as all its loves? If with the one, why not with the other? And why should a continuation of such a mutual love as our's be vouchsafed to us, if thousands are doomed to go wailing through Eternity, sorrowing for an unrequited or a hopelessly lost affection? Oh, Helen, Helen, why did we ever love?'

——

"'The death of Helen was so sudden, so unexpected, that I was nearly bereft of reason by the terrible blow. I was sometimes saddened by the thought of dying and leaving her alone in the world; but I had never thought of death in connection with Helen. It is ever thus in life. The evils we apprehend, and most dread, are seldom the ones that befall us. Fate seems to delight in taking us by surprise,— in tripping us suddenly into an unseen abyss.'

"'Oh, my lost, loved one! Is there anything in life that can compensate for the agonies I have suffered since your frail, inanimate form, was carried to the snow-covered graveyard? The world may be full of beauty, but I neither see nor enjoy it without you. Why was I permitted to love you with such an all-absorbing love that when you went out of life, you took with you all its sunshine and fragrance? Can it be that "He doeth all things well"? Though my lips give utterance to the expression, my heart refuses to acknowledge His goodness. Wherein lies the goodness of the blow that has so cruelly deprived me of my earthly treasure? Does such merciless sorrow as mine benefit me, or the world? Is it essential to the happiness of a future life that this one should be so full of suffering?'

---

"'This morning the preacher said, "It is a fearful thing to fall into the hands of the living God." What did he mean? Are we not always in His protecting arms? Can we ever fall out of His hands? Can it be that we slip from His grasp when death overtakes us? Banish the thought. I will not entertain it. The preacher did not realize what he said. It is a fearful thing to fall *out* of the hands of the living God. Only in His loving embrace do we rest secure.'

---

"'To-day I gaze down from the mountain tops upon a scene of surpassing loveliness. And I said, How many men and women within the whole scope of my vision are capable of enjoying this glorious prospect? Not the weary laborers who are toiling and sweating in the fields to earn a bare subsistence—not the wealthy possessors of all these farms and houses who are insanely struggling to amass more and more wealth—not the aimless wanderers, with blighted or broken lives, vainly endeavoring to flee away from their own wretchedness. A favored few there may be, perhaps, who enjoy it in a measure. But even they have no lease of happiness; and no security for their enjoyment continuing beyond the passing moment. Those who are happiest to-day may to-morrow be overwhelmed with grief as crushing and poignant as mine. Why then do we exist? Or why were we created? Why are the vast majority of the earth's inhabitants born only to struggle and toil, to suffer and weep? Can it be that we are born for a brief life, filled mostly with unhappiness, or at the best with only negative happiness, and that we then drop out of existence forever?'"

---

"Alice, I am charmed with the memoir," interrupted Emmeline, "but I think you have read enough for the present. Let us take a ramble."

---

"POMARIUS."

The delicate fruit-jelly spoken of on page 307 of this Journal, by the gifted authoress of "OUR SUMMER AT MAPLEWOOD," is now ready for the lovers of good things. It is, as "Cousin Kate" remarks, the filtered juice of sound, mellow, tart apples, concentrated to the last degree by careful evaporation. About ten pounds of apple-juice, clear and transparent as amber wine, is required to make one pound of this delicious extract. No sugar or gelatine is added, the careful processes of bruising the ripe apples, expressing the juice and filtering it, and then instantly concentrating it by a system of water-jacket evaporating pans, accomplishing the desired object. That object is to produce an agreeable and wholesome fruit-food, which shall keep forever in any climate, and which will produce a salutary effect upon the digestive organs. These results are attained by the Pomarius in perfection.

